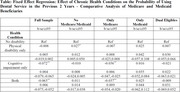# The Evaluation of Cognitive Impairment on Dental Care Utilization: A Comparative Analysis of Medicare and Medicaid Beneficiaries

**DOI:** 10.1002/alz70860_107690

**Published:** 2025-12-23

**Authors:** Zhang Zhang, Bei Wu

**Affiliations:** ^1^ Department of Health Policy and Management, Johns Hopkins Bloomberg School of Public Health, Baltimore, MD, USA; ^2^ New York University, New York, NY, USA

## Abstract

**Background:**

Dental care is critical for overall health and quality of life, yet barriers to access persist, particularly for individuals with disabilities. The objective of this study is to examine the relationship between cognitive impairment and dental care utilization, focusing on differential effects across health insurance types.

**Method:**

Using the Health and Retirement Study (HRS) from 1996 to 2018, we constructed a longitudinal sample cohort of 38,143 participants with 232, 018 observations. We adopted descriptive statistics and an individual fixed‐effect (F.E.) model to measure the relationship between cognitive impairment and the probability of using dental care in the past two years. Moreover, we examined the differential effect across Medicare and Medicaid Insurance groups by a heterogeneity analysis.

**Results:**

Among the observations, 41.4% reported no disability, 35.2% had physical disability only, 6.5% had cognitive impairment only, and 16.9% had both physical disability and cognitive impairment. Compared with no disability or physical disability, cognitive disability was significantly associated with reducing the probability of dental care utilization by 7.2 percentage points (*p* < 0.01, 95% CI: ‐0.079, ‐0.065), and cognitive impairment combined with physical disability was significantly associated with a reduction by 6.3 percentage points (*p* < 0.01, 95% CI: ‐0.075, ‐0.052). Moreover, heterogeneity analysis revealed that Medicare‐only beneficiaries experienced the largest decline in dental visits due to cognitive disability (3.6 percentage points, *p* < 0.01, 95% CI: ‐0.054, ‐0.020) in comparison to non‐Medicare/Medicaid, Medicaid only, and dual eligibles. However, the effects were statistically insignificant among Medicaid or dual‐eligible beneficiaries.

**Conclusion:**

This study reveals significant disparities in dental care utilization among individuals with cognitive disabilities, particularly among Medicare‐only beneficiaries. These findings underscore the urgent need for Medicare reforms to include supplemental benefits, such as dental coverage. Aligning dental coverage across Medicare and Medicaid is important to mitigate inequities, improve overall health outcomes, and reduce long‐term healthcare costs, particularly for vulnerable populations with disabilities.